# Animal model of simulated microgravity: a comparative study of hindlimb unloading via tail versus pelvic suspension

**DOI:** 10.1002/phy2.12

**Published:** 2013-06-12

**Authors:** Parimal Chowdhury, Ashley Long, Gabrielle Harris, Michael E Soulsby, Maxim Dobretsov

**Affiliations:** 1 Department of Physiology and Biophysics, University of Arkansas for Medical SciencesLittle Rock, Arkansas, 72205; 2 Department of Anesthesiology, University of Arkansas for Medical SciencesLittle Rock, Arkansas, 72205; 3 Department of Neurobiology and Developmental Sciences, University of Arkansas for Medical SciencesLittle Rock, Arkansas, 72205

**Keywords:** Hindlimb unloading, insulin, neuropathy, prediabetes, pressure hyperalgesia

## Abstract

The aim of this study was to compare physiological effects of hindlimb suspension (HLS) in tail- and pelvic-HLS rat models to determine if severe stretch in the tail-HLS rats lumbosacral skeleton may contribute to the changes traditionally attributed to simulated microgravity and musculoskeletal disuse in the tail-HLS model. Adult male Sprague-Dawley rats divided into suspended and control-nonsuspended groups were subjected to two separate methods of suspension and maintained with regular food and water for 2 weeks. Body weights, food and water consumption, soleus muscle weight, tibial bone mineral density, random plasma insulin, and hindlimb pain on pressure threshold (PPT) were measured. X-ray analysis demonstrated severe lordosis in tail- but not pelvic-HLS animals. However, growth retardation, food consumption, and soleus muscle weight and tibial bone density (decreased relative to control) did not differ between two HLS models. Furthermore, HLS rats developed similar levels of insulinopenia and mechanical hyperalgesia (decreased PPT) in both tail- and pelvic-HLS groups. In the rat-to-rat comparisons, the growth retardation and the decreased PPT observed in HLS-rats was most associated with insulinopenia. In conclusion, these data suggest that HLS results in mild prediabetic state with some signs of pressure hyperalgesia, but lumbosacral skeleton stretch plays little role, if any, in these pathological changes.

## Introduction

Hindlimb suspension (HLS) of rodents by the tail is a well-established approach to create a ground-based model of microgravity and musculoskeletal disuse that mimics many of the physiological changes associated with space flight, as well as with prolonged bed rest (Morey-Holton and Globus 2002; Morey-Holton et al. 2005; Carpenter et al. 2010). Among the most well-characterized changes in HLS rodents are the bone and muscle atrophy that are universally experienced by astronauts during space missions and by bed-ridden patients (Morey-Holton and Globus 2002; Graebe et al. 2004).

Tail-HLS in rodents is also a potential model for studies of another important effect of microgravity and disuse, specifically the development of a mild prediabetic state that is characterized by subclinical decrease in insulin secretion and loss of peripheral tissue sensitivity to insulin (Vernikos-Danellis et al. 1976; Stuart et al. 1988; Leach et al. 1991; Mikines et al. 1991; Tobin et al. 2002; Graebe et al. 2004). Within 1–2 weeks of tail-HLS, rats have been shown to develop insulinopenia (Nichols et al. 2008), insulin resistance and compensatory hyperinsulinemia (Stuart et al. 1993), or an increase in skeletal muscle insulin sensitivity (Henriksen et al. 1986; O'keefe et al. 2004). Although controversial, these observations warrant further exploration, as they may be critical for explaining limb disuse associated with bone loss as well as muscle atrophy (Stuart et al. 1993; Tischler et al. 1997; Thrailkill et al. 2005b; Fluckey et al. 2006; Wang et al. 2006). In addition, decreased insulin regulatory control has been linked to the development of muscle and visceral hyperalgesia in rat models of STZ-induced prediabetes (Romanovsky et al. 2010) and muscle pain in tail-HLS rats (Chowdhury et al. 2011). These problems may be related to both the back pain and gastrointestinal problems reported by a majority of astronauts and bed-ridden human patients (Styf et al. 2001). Furthermore, understanding limb immobilization-induced mechanical hyperalgesia in humans and animals (Hirose et al. 2001; Terkelsen et al. 2008; Ohmichi et al. 2012; Trierweiler et al. 2012), and pathogenesis of bone fracture and neuropathy risks in diabetes (Dobretsov et al. 2007; Leslie et al. 2007) provide additional rationale for this study.

Considering the nature of nociceptive changes in tail-HLS rats, we first tested the physical consequences of stretching the lumbosacral section of the spine during tail-HLS. Spine sprain and its associated injuries is one of most common causes of back pain in hospital admissions (Andersson 2008). Lengthening of gravity unloaded spinal columns and associated stretch of spinal roots have been discussed as a reason of low back pain in astronauts (Hutchinson et al. 1993; Styf et al. 2001; Carpenter et al. 2010). Signs of radiating low back pain were also described in SPARC-null mice model of lumbar intervertebral disc degeneration (Millecamps et al. 2012). Similarly the lumbosacral stretch of the spinal column is a likely contributor to the pathogenic picture in tail-HLS rat model of simulated microgravity. Whether this condition has any impact (affecting the pain and stress levels and possibly neurotrophic regulation) on the other major endpoints of hindlimb disuse in this model remain uncertain.

Therefore, the major focus of this work was to compare the effects of tail-HLS with those of a model that is less stressful on the lumbar spine, namely the model in which the hindlimbs are suspended at the pelvis by a belt, termed pelvic-HLS. We specifically compared these two HLS models with respect to their general stress levels (weight gain, daily water, and food intake), their pain on pressure threshold (PPT), of the extent of musculoskeletal atrophy and their plasma insulin levels following HLS, as a measure of pancreatic function.

## Materials and Methods

### Animals

All animal protocols were approved by Institutional Animal Care and Use committee and experiments were conducted in accord with the National Institute of Health Guide for the Care and Use of Laboratory Animals. Young adult male Sprague-Dawley rats (200–250 g, Harlan Inc., Indianapolis, IN) were used in all experiments. After 1 week of acclimation to the animal facilities and behavioral test regimen, rats were randomly assigned to control, tail- and pelvic-HLS groups (*n* = 6 or more per group). Experiments were performed in duplicate or triplicate. A total of 90 animals, 41 control, and 26 tail- and 23 belt HLS rats were studied. Body weights were measured at baseline and at 2 day intervals until euthanasia.

### Hindlimb suspension

Rats were suspended in individual plastic cages for 2 weeks at about 30 degrees head-down tilt. To set and adjust the suspension harness, rats were briefly (for 2–3 min) anesthetized by inhalation of isoflurane. After the animal had fully recovered from anesthesia, the angle of suspension was adjusted to make sure that when the animal is fully stretching its hindlimbs is unable to touch the ground.

#### Tail-HLS

Tail-HLS was conducted as described previously (Chowdhury and Soulsby 2008; Chowdhury et al. 2011) using a tail harness constructed by looping a strip of Skin-Trac orthopedic foam (Zimmer Inc., Charlotte, NC) around a pulley that can travel along a bar traversing the length of the cage. The adhesive surfaces of the reminder of the foam strip were applied to the long axis of the opposite sides of the tail, creating a “tail-sandwich,” secured by enwrapping it with orthopedic stockinet and three one-inch pieces of glass zip-reinforced strapping tape at the base, middle, and few centimeters from the tip of the tail.

#### Pelvic-HLS

**Figure 1:**
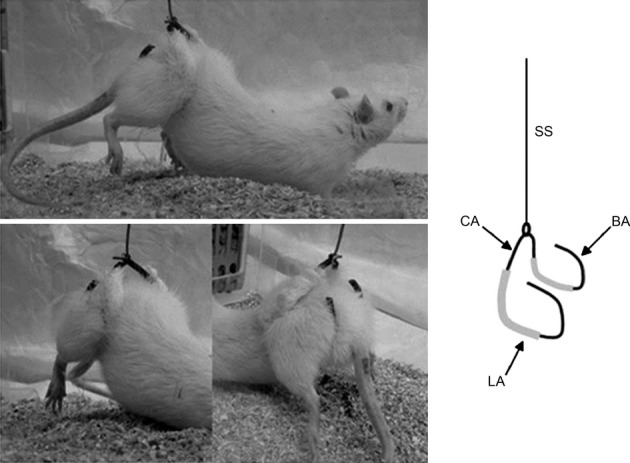
Pelvic hindlimb suspension technique and harness design. SS, suspension string; CA, central arc; LA, lower arm; BA, back arm.

The pelvic support harness was made out of thick insulated copper wire (core diameter 1.5 mm and outer diameter 4 mm) molded in its center into the hoop-like feature for securing a suspension string (SS) and bent as illustrated in the Figure 1 (right panel). The harness was adjusted to snugly, but without squeezing, follow the rat's body from the dorsum to the belly just in front of the rat's hips with lower arms (LA) of the harness fitting snuggly into the crease between the belly and respective inner thigh and folded back toward the main, central arc (CA) of the harness at the base of the tail following the rat's body curvature as closely as possible. In suspended position, the lower end of CA and front parts of LA provide most of the support to the rat's lower body against the force of gravity. Thus, these parts of the wire belt were wrapped with gauze-padded polyester tubing (7 mm OD) helping to distribute a pressure over a larger wire-body contact area, minimize chafing, and diminish possibility of rat body injury.

### PPT measurements

PPT values were determined at regular, 2–5 days intervals, with the final measurement being taken within 24 h of euthanasia. Dorsal hindlimb paw pressure pain withdrawal thresholds (PPT) were measured with a Randall–Selitto analgesia meter employing our standard laboratory technique (Romanovsky et al. 2006, 2010). Briefly, 10 determinations of PPT per animal (five per each hindlimb with an interval between sequential measurements greater than 10 min) were collected in each test session, filtered using mean ±1 SD cut-off, averaged for both limbs and expressed in grams. Threshold force of linearly increasing pressure (∼15 g/sec) was defined as a force that induces the first physical attempt of the animal to escape the stimulus. To avoid tissue injury the cut-off force was set to 250 g of pressure. HLS rats were temporarily unsuspended, with harness remaining in place, for only for the periods of behavioral test trials (2–3 min per trial). In addition, food and water consumption were measured daily during the period of suspension in subset of experiments.

On the last day of the experiment, animal was deeply anesthetized with a mixture of ketamine and acyl promazine (10:1 v/v), at a dose of 2 mL/kg of body weight and euthanized by cardiac bleeding.

### Bone mineral density measurements by dual energy X-ray absorptiometry

Longitudinal bone densitometry was performed, using the Piximus Bone Densitometer (Lunar Corp., Madison, WI), to obtain measurements of bone mineral density (BMD) and bone mineral content (BMC) from the lumbar spine and proximal right tibia of each animal as described previously (Fluckey et al. 2006). Scans were performed under anesthesia for attachment of the suspension device on day 0, the day HS (or control housing) began and on day 14 immediately prior to euthanasia. Subsequently, the L1–L4 spine measurements were combined comprising mostly of trabecular bone. The tibia scans were divided into the proximal and distal one thirds, and the diaphyseal midshaft one third for analysis, to account for the changes in cancellous and cortical bone densities, respectively, as previously described (Fluckey et al. 2006). The precision and accuracy of the Piximus instrument have been determined by repeated measurements of five animals, five times each. In-house precision analyses have been previously determined for adult rat femoral BMD to be 0.1% CV.

### Radiographic analysis of spines following HLS

To determine the extent to which HLS induced curvature of the spine, whole-body side X-ray images of deeply anesthetized rats after suspension and prior to euthanasia were taken using an AXR minishot 110 X-ray cabinet (Associated X-ray Corporation, East Haven, CT) at 3 mA, 33 kV for 20 sec using Kodak X-Omat TL film (Kodak, Rochester, NY) and processed on a Kodak X-Omat RP automated film processor.

### Blood collection and insulin analysis

Blood was collected by a ventricular puncture prior to euthanasia, plasma separated by centrifugation (5000*g*, 5 min), and stored at −20°C until further analysis of plasma Insulin levels using an Ultrasensitive Rat Insulin ELISA kit (Crystal Chem Inc., Downers Grove, IL), according to manufacturer's directions.

### Muscle analysis

Following euthanasia, the right leg soleus muscles were isolated, and their wet weight was measured and expressed in relative units (mg per kg of body weight).

### Statistical analysis

Statistical analysis was conducted using Statistica Software (StatSoft, Tulsa, OK) and Origin 9.0 Graphing and Analysis Software (OriginLab, Northampton, MA). The data were checked for normality of distribution (Shapiro–Wilk test) and analyzed using one- or two-way analysis of variance (ANOVA), followed by Tukey's or Bonferroni's post hoc comparison tests. Best fit analysis of frequency distributions was conducted using a Levenberg–Marguardt algorithm of χ^2^ minimization (Origin, Microcal, Northampton, MA). During the fitting procedure all independent parameters of the fitted Boltzmann functions were allowed to vary. Effects were considered as statistically significant at *P* < 0.05. Data in figures are expressed as mean ± SE.

## Results

### Radiographic analysis of the spine

**Figure 2:**
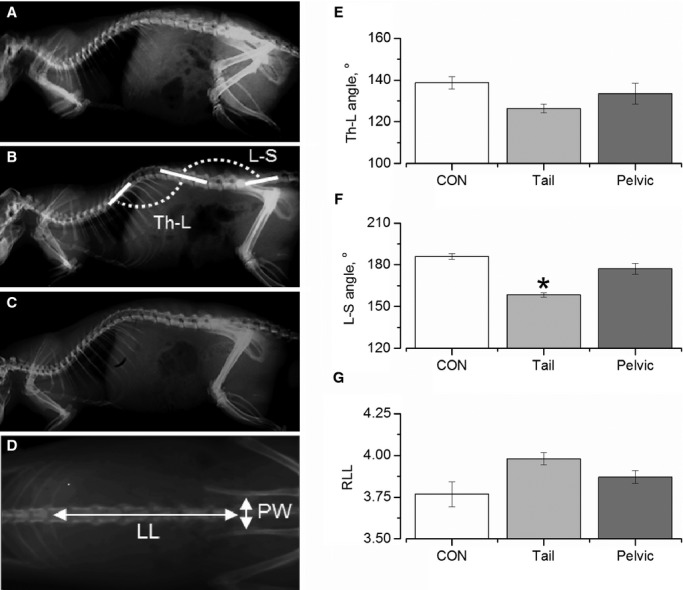
Effects of 2 weeks of tail- and pelvic-HLS on rat axial skeleton. (A–D) Representative X-ray images of, respectively, control and tail- and pelvic-HLS rats and DEXA image of thorax-sacrum region of the control rat spine are shown. Such images were used to determine effects of HLS on rat spine curvature by measuring a thoracolumbar (Th-L) and lumbosacral (L-S) angles as illustrated in (B) and relative lumbar length (RLL) as illustrated in (D). The Th-L and L-S angles, measure of kyphosis and lordosis, were defined as respective angles between Th_8-11_ and L_1-4_, and L_1-4_ and S_1-3_ segments of spinal column. (E–G). Mean values of, respectively, Th-L and L-S angles and relative lumbar length (RLL) measured in control (white bars), and tail- (light gray) and pelvic- (dark gray) HLS rats. Number of studied animals is 3, 6, and 6 for control, tail-HLS and pelvic-HLS groups in (E) and (F), and 3, 6, and 16 for respective groups in (G). One way ANOVA with Tukey post hoc test reveals statistically significant effect on rat axial skeleton of tail-HLS only and only with respect to L-S angle (F; *P < 0.05 for tail-HLS vs. control comparisons by Tukey test).

We hypothesized that the pelvic method of HLS would create less change in mechanical load on the rat's lumbosacral skeleton because of greatly expanded distribution of a gravity (compressive) load of the belt, compared with tensile load from the base of the tail in tail-HLS. To test this hypothesis, we used radiographic analysis by X-ray and dual energy X-ray absorptiometry (DEXA) images to measure and compare the thoracolumbar (Th-L) and lumbosacral (L-S) angles *(*analogous to Cobb's angles that measure a degree of kyphosis and lordosis in humans). The Th-L and L-S angles were defined as respective angles between Th_8-11_ and L_1-4_, and L_1-4_ and S_1-3_ segments of the spinal column (Fig. 2). In addition, the relative lumbar length (RLL) was measured between Th_13_ and S_1_ vertebrae, and normalized to pelvic girdle width (PW) measured at S_1_ vertebral level. The RLL and Th-L angles were comparable between control and tail- and pelvic-HLS rats (Fig. 2). However, supporting the concern regarding the deteriorating effect of tail-HLS on the state of the rat axial skeleton, tail (but not pelvic)-HLS was shown to result in a statistically significant decrease in the L-S angle (Fig. 2F).

### General characteristics (food, water, weight)

**Figure 3:**
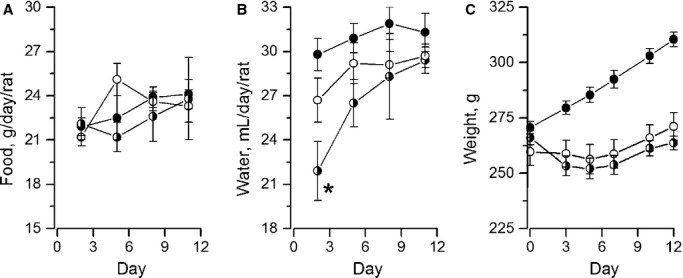
Effects of 2 weeks of tail- and pelvic-HLS on the rat food (A) and water (B) consumption and weight gain (C). (A) Daily food consumption during a suspension period by control, tail-, and pelvic-HLS rats (*n* = 13, 6, and 12 animals per group, respectively. No between-group differences is detected by two-way repeated measures (RM) ANOVA at any of postsuspension day (*F*(2,84) = 0.44, *P* = 0.651). (B) Daily water consumption during a suspension period by control, tail-, and pelvic-HLS rats (*n* = 13, 6, and 12 animals per group, respectively). In the beginning of suspension period tail-HLS rats consume less food than either control or pelvic-HLS rats (asterisk; two-way RM ANOVA: *F*(2,84) = 3.50, *P* = 0.045; Bonferroni test: *P* < 0.01). (C) Body weight at baseline (day 0) and during suspension by control, tail-, and pelvic-HLS groups (*n* = 39, 18, and 21 animals per group, respectively). HLS results in net weight loss in tail-HLS (*P* < 0.05; days 3–7 vs. day 0; Tukey test) and in growth retardation in pelvic-HLS groups of animals. Later in experiment animal's weight gain by HLS rats resumes but control-HLS rats weight differences persist through entire 2 weeks of HLS period (between-group comparison by two-way RM ANOVA, followed by Bonferroni test; within-group comparisons by one-way RM ANOVA followed by Tukey test). All panels: closed, half-closed, and opened circles represent control, tail-HLS, and pelvic-HLS groups of rats, respectively.

Changes in weight and food and water intake are indirect indicators of stress experienced by experimental animals. In our experiments, we did not observe any difference in food consumption between any of the groups of rats studied (Fig. 3A), although tail-HLS demonstrated a slight but significant decrease in water consumption relative to control in the beginning of suspension period, Fig. 3B). Both HLS groups experienced statistically significant growth retardation. In fact, tail-HLS rats even lost some weight (about 13 g) during first 3 days of HLS. Weight gain by suspended animals resumed during the second week of suspension but remained slower than that in control rats (Fig. 3C).

### Muscle atrophy and bone loss

**Figure 4:**
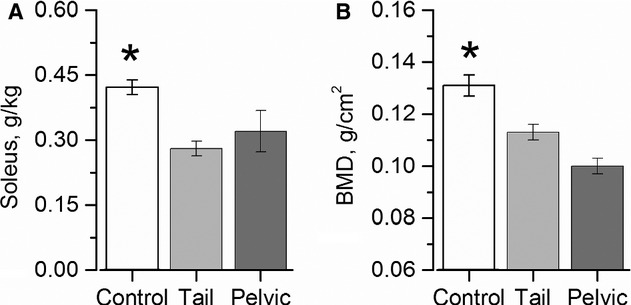
Soleus muscle weight (A) and tibial bone mineral density (B) of control and hindlimb suspended rats. (A) Mean relative weight of soleus muscle of age-matched control, tail-, and pelvic-HLS rats (*n* = 12, 4, and 15 animals per group, respectively). Differences between control and tail- or pelvic-HLS groups are significant at *P* < 0.01 by one-way ANOVA with post hoc Tukey test. (B) Mean tibial bone mineral density (BMD) of age-matched control, tail-, and pelvic-HLS rats (*n* = 13, 12, and 8 animals per group, respectively). Differences between control and tail- or pelvic-HLS groups are significant at *P* < 0.01 by one-way ANOVA with post hoc Tukey test.

Disuse associated muscle atrophy and bone loss represent hallmark effects of lower limb musculoskeletal disuse in human bed-ridden patients and space mission participants (Morey-Holton and Globus 2002; Graebe et al. 2004). These changes are also well-established consequences of rat HLS models (Morey-Holton and Globus 2002; Morey-Holton et al. 2005; Carpenter et al. 2010). In our experiments, we observed that both soleus muscle weight and tibial cancellous bone mineral density were decreased to the same degree after 2 weeks of either tail or pelvic suspension (Fig. 4). As an internal control, however, no changes in humerous density was detected in either of the HLS models during an experiment in the remaining loaded limbs (data not shown).

### PPT and random plasma insulin concentration

**Figure 5:**
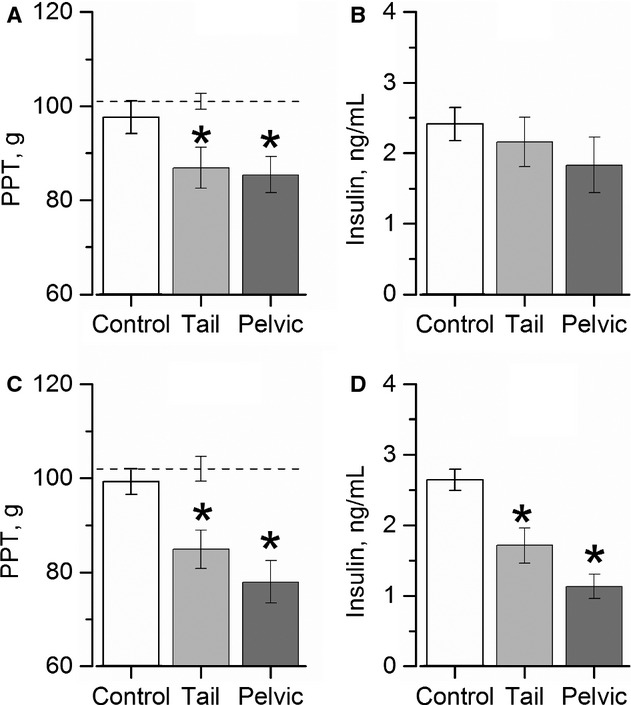
Pain on pressure threshold (A and C) and random plasma insulin concentration (B and D) measured in control and hindlimb suspended rats. (A) Pain on pressure thresholds (PPTs) measured at baseline (*n* = 90; horizontal dashed line) and after 2 weeks of the experiment in control and tail- and pelvic-HLS rats (*n* = 41, 26, and 23 animals per group, respectively). Both tail- and pelvic-HLS groups are statistically significantly different from the baseline PPT level (*P* < 0.01 by one-way ANOVA with post hoc Tukey test, asterisks). (B) Random plasma insulin concentration of control, tail-, and pelvic-HLS rats (*n* = 31, 15, and 17 animals per group, respectively). Between-group differences are not significant by one-way ANOVA with post hoc Tukey test. (C) Pain on pressure thresholds (filtered data, see text for the procedure) measured at baseline (*n* = 40; horizontal dashed line) and after 2 weeks of experiment in control and tail- and pelvic-HLS rats (*n* = 19, 8, and 13 animals per group, respectively). Both tail- and pelvic-HLS groups are statistically significantly different from both control and the experiment entry PPT levels (*P* < 0.01 by one-way ANOVA with post hoc Tukey test, asterisks). (D) Random plasma insulin concentration (filtered data, see text for the procedure) after 2 weeks of the experiment in control and tail- and pelvic-HLS rats (*n* = 19, 8, and 13 animals per group, respectively). Tail- and pelvic-HLS groups are statistically significantly different from control group (*P* < 0.01 by one-way ANOVA with post hoc Tukey test, asterisks). Filtering did not result in statistically significant changes of mean values of either PPT or insulin level in any of groups of animals (control, tail- or pelvic-HLS; *P* > 0.05, one-way ANOVA followed by Tukey test).

To date, nociceptive changes have received relatively little attention in the HLS model. Recently, however, we provided the evidence of pressure hyperalgesia developing in tail-HLS rats, as well as an indication that this could be associated with insulinopenia (Chowdhury et al. 2011). Here, we determined whether the PPT and associated insulinopenia was comparable in the tail-HLS and the pelvic-HLS animals. In these experiments, pressure hyperalgesia (decrease in PPT) developed to a similar degree after 2 weeks of either tail or pelvic suspension (Fig. 5A). Mean insulin measurements, however, demonstrated only a tendency to decrease from the control, to tail-HLS and further to pelvic-HLS groups of rats (Fig. 5B). Both PPT and insulin measurements are prone to high variability. This is specifically so for random insulin measures (fasted insulin was not analyzed as it may not represent conditions under which PPT was measured), considering that we did not control the time elapsed from the rat that was last eating prior to blood sampling. To circumvent this problem, we reanalyzed the data using the following approach. First, the PPT and insulin data that were collected at the end of experiment were filtered for outliers using mean ± 1 SD rule for each group of animals (control, tail-, and pelvic-HLS) separately. Then animals with both PPT and insulin measurements passing the filter test were selected for further study.

**Figure 6:**
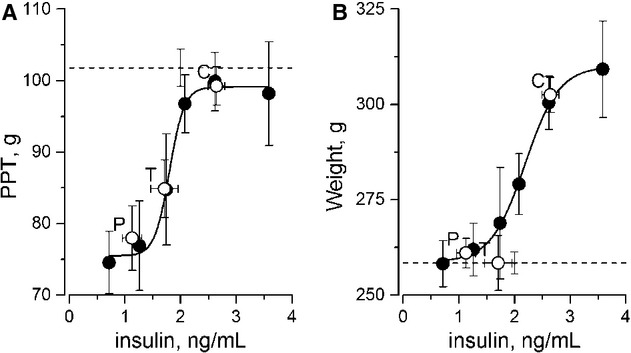
Insulin-PPT (A) and insulin-rat weight (B) relationships in control and HLS experiments. In both panels (the same as used in Fig. 5C and D) dataset of 40 animals was used. Filled circles represent mean PPT (A) and weight (B) values measured in groups of rats having random insulin levels within 0–1.0, 1.01–1.5, 1.51–2, 2.01–2.25, 2.26–3.00, and 3.01–4.0 ng/mL ranges (9, 4, 4, 9, 9, and 5 rats per respective range). Open circles represent mean characteristics of control and tail- and pelvic-HLS groups (19, 8, and 13 rats, “C,” “T,” and “P” text labels, respectively). Dashed lines represent mean baseline PPT (A) and weight (B) for all 40 rats studied. Solid curves are result of best fit of the Boltzmann equation (*y* = A2 + (A1 − A2)/(1 + exp([x − x0]/dx)) to the data shown by filled circles. The fit parameters are A1 = 75.0 ± 1.0 g, A2 = 99.1 ± 1.0 g, x0 = 1.80 ± 0.04 ng/mL, and dx = 0.13 ± 0.04 ng/mL (A: χ^2^ = 1.739, adjusted *R*^2^ = 0.986) and A1 = 259 ± 1 g, A2 = 310 ± 1 g, x0 = 2.19 ± 0.03 ng/mL, and dx = 0.31 ± 0.03 ng/mL (B: χ^2^ = 1.346, adjusted *R*^2^ = 0.997).

Analysis of filtered data measured in this way have confirmed previous findings (Chowdhury et al. 2011) of decreased PPT in groups of tail-HLS animals, and demonstrate for the first time that both tail- and pelvic-HLS rats have similarly decreased PPT (relative to control and relative to baseline PPT level, asterisks in Fig. 5C). Furthermore, this analysis revealed statistically significant differences of plasma insulin levels at euthanasia between control and either tail- or pelvic-HLS rats (Fig. 5D). Finally, further analysis of insulin levels categorized into ranges of plasma concentrations suggested the existence of sigmoidal dose–response relationships between the plasma insulin level and PPT and body weight of studied animals, which could explain, in part, both the decrease in PPT and the prevention of weight gain in HLS rats (Fig. 6A and B, respectively).

## Discussion

This work confirms the results of previous studies (reviewed in Morey-Holton et al. 2005) regarding the deleterious effects of short-term HLS on body weight and postural muscle weight, and hindlimb bone mineral density. In addition, these findings confirm the recent observation (Chowdhury et al. 2011) of decreasing PPT of the hindlimb paw in HLS rats. The novel observations of this study are that despite sharply different effects of tail- and pelvic-HLS techniques on mechanical loads experienced by the lower spine of the rat, the above mentioned effects of HLS developed similarly in both models. Another important result of our study is that it extends evidence for the development of subclinical insulinopenia in HLS animals (Hamburg et al. 2007; Chowdhury et al. 2011) with a possible link of this prediabetic state to the development of deep pressure hyperalgesia.

The tail-HLS model is well a well-established model of simulated microgravity (Morey-Holton and Globus 2002; Morey-Holton et al. 2005). The problem of lordosis (Fig. 2) in this model has been recognized for some time (Templeton et al. 1984; Wronski and Morey-Holton 1987). However, it has not posed significant challenges prior to the measurement of pain thresholds being studied in tail-HLS animals. Our finding of pressure hyperalgesia developing in tail-suspended rats (Chowdhury et al. 2011) warranted a more detailed analysis of this issue, as lumbar stretch (increased tensile mechanical load to the spine) is considered as one of possible causes of low back pain in astronauts and bed-ridden patients (Hutchinson et al. 1993; Styf et al. 2001; Carpenter et al. 2010). To address this we have compared here the tail- and newly developed pelvic-suspension models of HLS.

Considering the purely technical aspects, when tail-HLS was compared to the procedure of pelvic suspension, it is more labor consuming in its preparative period (pelvic belt manufacturing), but easier with regard to suspension procedure itself (placing the belt vs. tail harness) and has about the same success rate as measured by the duration of the rat remaining suspended through the entire 2 weeks of experiment. Only about 5% of rats in each group needed to be removed from experiment as repeatedly (more than once) requiring their harness to be reattached. By its design, the pelvic-HLS does not carry the problem of tail harness placement – associated risk of impaired tail blood flow. However, we recognize that the state of pelvic belt support arm padding must be paid close attention to with wet or damaged padding being immediately replaced. None of pelvic-HLS rats needed to be removed from these experiments because of inner thigh skin inflammation at the harness contact points. In our view, however, development of such inflammation may become a real problem in the experiments with longer than 2 weeks of suspension period.

Our current study demonstrated clearly different effects of the two suspension methods with regard to spinal lumbosacral curvature, which was more pronounced in the tail-HLS than the pelvic-HLS (Fig. 2). However, most of the other measured endpoints behaved similarly between two models of HLS. The exception was a decrease in water intake and net weight loss observed in the beginning of HLS period in tail-HLS but not in pelvic-HLS rats (Fig. 3B and C) suggesting a higher stress level in the tail-HLS group (see Jacobsen et al. 2012). These differences, however, were transient. By the end of experiment, weight gain, food consumption, bone mineral density, soleus muscle mass, circulating insulin, or PPT levels were not distinguishable between two HLS models. Thus, we found no evidence of lumbosacral axial skeleton stretch as a factor in the pathologic etiology that develops during 2 weeks of HLS in the tail-HLS rat model. However, it cannot be excluded that such tensile stretch might contribute to pathology in experiments with longer period of tail-HLS.

The musculoskeletal deficiency of HLS rats in our study is comparable to that reported for tail-HLS animals studied for the same time period (2 weeks of HLS) by other groups. Specifically, soleus muscle of our HLS rats weighted about 40% less than soleus muscle of age-matched control rats (Fig. 4A), which is well in the range of 25–49% loss reported for male Wistar or SD rats soleus muscle after 2 weeks of tail-HLS (Templeton et al. 1984; Somody et al. 1998; Picquet and Falempin 2003; Fujino et al. 2005; Mueller et al. 2005; Zhang et al. 2009).Our observed ∼20% difference in bone mineral density in the proximal tibia of control and HLS rats (Fig. 4B) is also within the range of 8–21% effect of tail-HLS on rat proximal tibia found in the literature (Bloomfield et al. 2002; Allen et al. 2006; Swift et al. 2010). Exact mechanisms of these effects of limb unloading are not known. It is tempting to speculate, however, that at least in part they may be associated with weakened activity of insulin/insulin-like growth factor–1 (IGF-1) axis in suspended rats. Both insulin and IGF-1 are well known regulators of protein synthesis, bone, and muscle mass development (Davis et al. 1998; Thrailkill et al. 2005b; Han et al. 2007). Dysfunction of this control, associated with either decrease in circulating concentrations or peripheral insulin/IGF-1 resistance occurs in healthy humans during space flight or prolonged bed rest and physical inactivity (Dolkas and Greenleaf 1977; Mikines et al. 1991; Tobin et al. 2002; Hamburg et al. 2007; Cree et al. 2010), and in skeletal muscles of tail-HLS rats (Mondon et al. 1992; Stuart et al. 1993; Han et al. 2007). In the latter model, the decrease in circulating insulin (Nichols et al. 2008) and IGF-1 (Perrien et al. 2007) was detected previously and confirmed with regard to insulin by us recently (Chowdhury et al. 2011) and in this study (Fig. 5). Our data also suggested existence of close relationships between the rat's body weight gain and random plasma insulin concentration, with 2.2 ng/mL of insulin as concentration required to achieve half-maximal stimulation of weight gain (Fig. 5B). Interestingly, this value is very close to the 2.3 ng/mL insulin concentration required for half-maximal activation of whole body amino acid disposal in young pigs (Davis et al. 1998). Another line of indirect support to the role of the insulin/IGF-1 axis in the pathogenesis of musculoskeletal disuse conditions is that normal activity of this axis was shown as critical to keep the rate of protein degradation by ubiquitin-proteasome pathway at a low level (Tischler et al. 1997; Wang et al. 2006). Hyperactivation of this pathway was identified as a cause of degradation of rat myosin heavy chain in muscles of astronauts (space shuttle flight [STS-90]; Ikemoto et al. 2001). Accelerated protein degradation can also explain weight gain retardation, despite the preserved food intake in our HLS experiments (Fig. 3). Finally, muscle protein degradation and muscle wasting is accelerated in mouse models of insulin resistance and type 2 diabetes (Wang et al. 2006). Similarly, bone formation is impaired in rat and mouse models of type 1 and type 2 diabetes (Thrailkill et al. 2005a; Liu et al. 2007).

With regard to diabetes, our data provide further evidence demonstrating the remarkable phenotypic resemblance between HLS rats and the rat model of streptozotocin-induced early type 1 prediabetes (STZ-PD; Romanovsky et al. 2006, 2010). In both models, there is a moderate decrease in circulating insulin, which is not associated with changes in glucose metabolism (plasma glucose was not measured in this study, but was found normal in tail-HLS rats in our previous work; Chowdhury et al. 2011), but appeared closely correlating with a decrease in PPT measured in same animals. “Insulin-PPT” relationships could be fit with a sigmoidal curve in both models, except that best fit ED50 for an “insulin-PPT” relationship to maintain normal pain threshold was lower in STZ- than in HLS-rats (1.35 ± 0.06 vs. 1.8 ± 0.04 ng/mL, respectively; Fig. 5 in Romanovsky et al. 2006 and Fig. 6A, this work). Another difference between these models is weight gain, which was preserved at normal level in STZ-PD, but suppressed in proportion to a degree of insulinopenia in animals in this study. The exact reason for these differences remains to be determined. They may, however, reflect differences of the models with respect to systemic insulin resistance. STZ-PD rats have normal glucose tolerance (Romanovsky et al. 2004) demonstrating no signs of loss of skeletal muscle insulin sensitivity. The possibility of development of muscle insulin resistance in tail-HLS rats was not addressed in this study. However, it has been reported in the literature (Mondon et al. 1992; Stuart et al. 1993; Han et al. 2007). If present in our HLS rats, it could explain higher insulin requirements for maintaining normal growth and PPT level seen in moderately insulinopenic and insulin resistant (HLS) rats as compared to insulinopenic but insulin sensitive STZ-PD animals.

Tentative insulin dependence of evoked PPT observed in both STZ and HLS normoglycemic rats constitute an important issue on its own. Musculoskeletal pain is one of most prevalent symptoms of diabetic neuropathy (Dobretsov et al. 2010), which as most of other symptoms of this neuropathy, was traditionally attributed to neurotoxicity of diabetic hyperglycemia (Tomlinson and Gardiner 2008). The studies above clearly indicate that at least in some cases a direct effect of impaired insulin signaling, but not hyperglycemia, may serve as a pathogenic substrate for hyperactivity of deep muscle nociceptors. In humans, acute unloading the bodyweight results in a general increase of the withdrawal reflex excitability to prepare the limb in taking the first walking step (Serrao et al. 2012). This mechanism is, however, unlikely to add to pressure hyperalgesia in HLS rats, since it starts to develop only after about a week of HLS (Chowdhury et al. 2011). In a recent study, blood corticosterone level and hindlimb paw hypersensitivity to mechanical (von Frey hair) stimuli were observed increasing similarly in HLS rats and in rats that had tail suspension device attached but were not suspended (Tanaka et al. 2013). The authors had concluded that it is not microgravity or inactivity, but a restraint stress constitutes a major pathogenic factor determining changes in mechanical withdrawal thresholds in HLS-rat. Although von Frey filament and paw pressure tests measure activity of different nociceptive pathways (skin vs. deep muscle nociceptors) and the relations between cortisol levels and nociception are not straightforward (Taylor et al. 1998; Benedetti et al. 2012; Jacobsen et al. 2012), our data do not allow us to exclude the possibility above. Further studies are needed to clarify the mechanisms of observed PPT changes and to establish their relevance (if any) to low back pain as a common comorbidity of space flight and prolonged bed rest in humans (Hutchinson et al. 1993; Styf et al. 2001; Carpenter et al. 2010).

In conclusion, these data show that HLS (by either tail- or pelvic-suspension method) results in delay in the weight gain, skeleton–muscular deficiencies, and a mild prediabetic state with signs of exaggeration of evoked deep muscle pain. However, the increased tensile load on the lumbosacral spine in tail-HLS plays little if any role in these pathological changes. Pelvic suspension appears as a suitable alternative to the tail-HLS method in experiments with short-term (2 weeks) rat hindlimb unloading. It remains to be determined if longer periods of HLS are sustainable with this technique.

## References

[b1] Allen MR, Hogan HA, Bloomfield SA (2006). Differential bone and muscle recovery following hindlimb unloading in skeletally mature male rats. J. Musculoskelet. Neuronal Interact.

[b2] Andersson G (2008). Burden of musculoskeletal diseases in the United States: prevalence, societal and economic cost.

[b3] Benedetti M, Merino R, Kusuda R, Ravanelli MI, Cadetti F, dos Santos P (2012). Plasma corticosterone levels in mouse models of pain. Eur. J. Pain.

[b4] Bloomfield SA, Allen MR, Hogan HA, Delp MD (2002). Site- and compartment-specific changes in bone with hindlimb unloading in mature adult rats. Bone.

[b5] Carpenter RD, Lang TF, Bloomfield SA, Bloomberg JJ, Judex S, Keyak JH (2010). Effects of long-duration spaceflight, microgravity, and radiation on the neuromuscular, sensorimotor, and skeletal systems. J. Cosmol.

[b6] Chowdhury P, Soulsby M (2008). Oxidant/anti-oxidant status in rats exposed to simulated weightlessness by hind-limb unloading and reloading. Open Clin. Chem. J.

[b7] Chowdhury P, Soulsby M, Jayroe J, Akel NS, Gaddy D, Dobretsov M (2011). Pressure hyperalgesia in hind limb suspended rats. Aviat. Space Environ. Med.

[b8] Cree MG, Paddon-Jones D, Newcomer BR, Ronsen O, Aarsland A, Wolfe RR (2010). Twenty-eight-day bed rest with hypercortisolemia induces peripheral insulin resistance and increases intramuscular triglycerides. Metabolism.

[b9] Davis TA, Burrin DG, Fiorotto ML, Reeds PJ, Jahoor F (1998). Roles of insulin and amino acids in the regulation of protein synthesis in the neonate. J. Nutr.

[b10] Dobretsov M, Romanovsky D, Stimers JR (2007). Early diabetic neuropathy: triggers and mechanisms. World J. Gastroenterol.

[b11] Dobretsov M, Backonja M, Romanovsky D, Stimers JR, Ma C, Zhang J-M (2010). Animal models of diabetic neuropathic pain. Animal models of pain.

[b12] Dolkas CB, Greenleaf JE (1977). Insulin and glucose responses during bed rest with isotonic and isometric exercise. J. Appl. Physiol.

[b13] Fluckey JD, Knox M, Smith L, Dupont-Versteegden EE, Gaddy D, Tesch PA (2006). Insulin-facilitated increase of muscle protein synthesis after resistance exercise involves a MAP kinase pathway. Am. J. Physiol. Endocrinol. Metab.

[b14] Fujino H, Kohzuki H, Takeda I, Kiyooka T, Miyasaka T, Mohri S (2005). Regression of capillary network in atrophied soleus muscle induced by hindlimb unweighting. J. Appl. Physiol.

[b15] Graebe A, Schuck EL, Lensing P, Putcha L, Derendorf H (2004). Physiological, pharmacokinetic, and pharmacodynamic changes in space. J. Clin. Pharmacol.

[b16] Hamburg NM, McMackin CJ, Huang AL, Shenouda SM, Widlansky ME, Schulz E (2007). Physical inactivity rapidly induces insulin resistance and microvascular dysfunction in healthy volunteers. Arterioscler. Thromb. Vasc. Biol.

[b17] Han B, Zhu MJ, Ma C, Du M (2007). Rat hindlimb unloading down-regulates insulin like growth factor-1 signaling and AMP-activated protein kinase, and leads to severe atrophy of the soleus muscle. Appl. Physiol. Nutr. Metab.

[b18] Henriksen EJ, Tischler ME, Johnson DG (1986). Increased response to insulin of glucose metabolism in the 6-day unloaded rat soleus muscle. J. Biol. Chem.

[b19] Hirose M, Kaneki M, Sugita H, Yasuhara S, Ibebunjo C, Martyn JA (2001). Long-term denervation impairs insulin receptor substrate-1-mediated insulin signaling in skeletal muscle. Metabolism.

[b20] Hutchinson K, Hargens AR, Murthy G, Watenpaugh DE, Convertino VA, Wing PC (1993). Six degrees head-down tilt as a back pain model for actual microgravity. FASEB J.

[b21] Ikemoto M, Nikawa T, Takeda S, Watanabe C, Kitano T, Baldwin KM (2001). Space shuttle flight (STS-90) enhances degradation of rat myosin heavy chain in association with activation of ubiquitin-proteasome pathway. FASEB J.

[b22] Jacobsen KR, Kalliokoski O, Teilmann AC, Hau J, Abelson KS (2012). Postsurgical food and water consumption, fecal corticosterone metabolites, and behavior assessment as noninvasive measures of pain in vasectomized BALB/c mice. J. Am. Assoc. Lab. Anim. Sci.

[b23] Leach CS, Cintron NM, Krauhs JM (1991). Metabolic changes observed in astronauts. J. Clin. Pharmacol.

[b24] Leslie WD, Lix LM, Prior HJ, Derksen S, Metge C, O'Neil J (2007). Biphasic fracture risk in diabetes: a population-based study. Bone.

[b25] Liu Z, Aronson J, Wahl EC, Liu L, Perrien DS, Kern PA (2007). A novel rat model for the study of deficits in bone formation in type-2 diabetes. Acta Orthop.

[b26] Mikines KJ, Richter EA, Dela F, Galbo H (1991). Seven days of bed rest decrease insulin action on glucose uptake in leg and whole body. J. Appl. Physiol.

[b27] Millecamps M, Tajerian M, Naso L, Sage EH, Stone LS (2012). Lumbar intervertebral disc degeneration associated with axial and radiating low back pain in ageing SPARC-null mice. Pain.

[b28] Mondon CE, Rodnick KJ, Dolkas CB, Azhar S, Reaven GM (1992). Alterations in glucose and protein metabolism in animals subjected to simulated microgravity. Adv. Space Res.

[b29] Morey-Holton ER, Globus RK (2002). Hindlimb unloading rodent model: technical aspects. J. Appl. Physiol.

[b30] Morey-Holton E, Globus RK, Kaplansky A, Durnova G (2005). The hindlimb unloading rat model: literature overview, technique update and comparison with space flight data. Adv. Space Biol. Med.

[b31] Mueller PJ, Foley CM, Hasser EM (2005). Hindlimb unloading alters nitric oxide and autonomic control of resting arterial pressure in conscious rats. Am. J. Physiol. Regul. Integr. Comp. Physiol.

[b32] Nichols KR, Chowdhury P, Dupont-Vesteegden EE (2008). Pancreatic response to hind limb suspension in rats is affected by age. Open Clin. Chem. J.

[b33] Ohmichi Y, Sato J, Ohmichi M, Sakurai H, Yoshimoto T, Morimoto A (2012). Two-week cast immobilization induced chronic widespread hyperalgesia in rats. Eur. J. Pain.

[b34] O'keefe MP, Perez FR, Sloniger JA, Tischler ME, Henriksen EJ (2004). Enhanced insulin action on glucose transport and insulin signaling in 7-day unweighted rat soleus muscle. J. Appl. Physiol.

[b35] Perrien DS, Akel NS, Dupont-Versteegden EE, Skinner RA, Siegel ER, Suva LJ (2007). Aging alters the skeletal response to disuse in the rat. Am. J. Physiol. Regul. Integr. Comp. Physiol.

[b36] Picquet F, Falempin M (2003). Compared effects of hindlimb unloading versus terrestrial deafferentation on muscular properties of the rat soleus. Exp. Neurol.

[b37] Romanovsky D, Hastings SL, Stimers JR, Dobretsov M (2004). Relevance of hyperglycemia to early mechanical hyperalgesia in streptozotocin-induced diabetes. J. Peripher. Nerv. Syst.

[b38] Romanovsky D, Cruz NF, Dienel GA, Dobretsov M (2006). Mechanical hyperalgesia correlates with insulin deficiency in normoglycemic streptozotocin-treated rats. Neurobiol. Dis.

[b39] Romanovsky D, Wang J, Al Chaer ED, Stimers JR, Dobretsov M (2010). Comparison of metabolic and neuropathy profiles of rats with streptozotocin-induced overt and moderate insulinopenia. Neuroscience.

[b40] Serrao M, Spaich EG, Andersen OK (2012). Modulating effects of bodyweight unloading on the lower limb nociceptive withdrawal reflex during symmetrical stance. Clin. Neurophysiol.

[b41] Somody L, Fagette S, Blanc S, Frutoso J, Gharib C, Gauquelin-Koch G (1998). Regional blood flow in conscious rats after head-down suspension. Eur. J. Appl. Physiol. Occup. Physiol.

[b42] Stuart CA, Shangraw RE, Prince MJ, Peters EJ, Wolfe RR (1988). Bed-rest-induced insulin resistance occurs primarily in muscle. Metabolism.

[b43] Stuart CA, Kidder LS, Pietrzyk RA, Klein GL, Simmons DJ (1993). Rat tail suspension causes a decline in insulin receptors. Exp. Toxicol. Pathol.

[b44] Styf JR, Hutchinson K, Carlsson SG, Hargens AR (2001). Depression, mood state, and back pain during microgravity simulated by bed rest. Psychosom. Med.

[b45] Swift JM, Nilsson MI, Hogan HA, Sumner LR, Bloomfield SA (2010). Simulated resistance training during hindlimb unloading abolishes disuse bone loss and maintains muscle strength. J. Bone Miner. Res.

[b46] Tanaka Y, Nakano J, Hamaue Y, Sekino Y, Sakamoto J, Kataoka H (2013). Hindlimb suspension does not influence mechanical sensitivity, epidermal thickness, and peripheral nerve density in the glabrous skin of the rat hind paw. Physiol. Res.

[b47] Taylor BK, Akana SF, Peterson MA, Dallman MF, Basbaum AI (1998). Pituitary-adrenocortical responses to persistent noxious stimuli in the awake rat: endogenous corticosterone does not reduce nociception in the formalin test. Endocrinology.

[b48] Templeton GH, Padalino M, Manton J, Glasberg M, Silver CJ, Silver P (1984). Influence of suspension hypokinesia on rat soleus muscle. J. Appl. Physiol.

[b49] Terkelsen AJ, Bach FW, Jensen TS (2008). Experimental forearm immobilization in humans induces cold and mechanical hyperalgesia. Anesthesiology.

[b50] Thrailkill KM, Liu L, Wahl EC, Bunn RC, Perrien DS, Cockrell GE (2005a). Bone formation is impaired in a model of type 1 diabetes. Diabetes.

[b51] Thrailkill KM, Lumpkin CK, Bunn RC, Kemp SF, Fowlkes JL (2005b). Is insulin an anabolic agent in bone? Dissecting the diabetic bone for clues. Am. J. Physiol. Endocrinol. Metab.

[b52] Tischler ME, Satarug S, Aannestad A, Munoz KA, Henriksen EJ (1997). Insulin attenuates atrophy of unweighted soleus muscle by amplified inhibition of protein degradation. Metabolism.

[b53] Tobin BW, Uchakin PN, Leeper-Woodford SK (2002). Insulin secretion and sensitivity in space flight: diabetogenic effects. Nutrition.

[b54] Tomlinson DR, Gardiner NJ (2008). Glucose neurotoxicity. Nat. Rev. Neurosci.

[b55] Trierweiler J, Gottert DN, Gehlen G (2012). Evaluation of mechanical allodynia in an animal immobilization model using the von Frey method. J. Manipulative Physiol. Ther.

[b56] Vernikos-Danellis J, Leach CS, Winget CM, Goodwin AL, Rambaut PC (1976). Changes in glucose, insulin, and growth hormone levels associated with bedrest. Aviat. Space Environ. Med.

[b57] Wang X, Hu Z, Hu J, Du J, Mitch WE (2006). Insulin resistance accelerates muscle protein degradation: activation of the ubiquitin-proteasome pathway by defects in muscle cell signaling. Endocrinology.

[b58] Wronski TJ, Morey-Holton ER (1987). Skeletal response to simulated weightlessness: a comparison of suspension techniques. Aviat. Space Environ. Med.

[b59] Zhang R, Bai YG, Lin LJ, Bao JX, Zhang YY, Tang H (2009). Blockade of AT1 receptor partially restores vasoreactivity, NOS expression, and superoxide levels in cerebral and carotid arteries of hindlimb unweighting rats. J. Appl. Physiol.

